# Prevalence of *Salmonella* and *Listeria monocytogenes* in non-traditional irrigation waters in the Mid-Atlantic United States is affected by water type, season, and recovery method

**DOI:** 10.1371/journal.pone.0229365

**Published:** 2020-03-17

**Authors:** Manan Sharma, Eric T. Handy, Cheryl L. East, Seongyun Kim, Chengsheng Jiang, Mary Theresa Callahan, Sarah M. Allard, Shirley Micallef, Shani Craighead, Brienna Anderson-Coughlin, Samantha Gartley, Adam Vanore, Kalmia E. Kniel, Joseph Haymaker, Rico Duncan, Derek Foust, Chanelle White, Maryam Taabodi, Fawzy Hashem, Salina Parveen, Eric May, Anthony Bui, Hillary Craddock, Prachi Kulkarni, Rianna T. Murray, Amy R. Sapkota

**Affiliations:** 1 United States Department of Agriculture, Agricultural Research Service, Northeast Area, Environmental Microbial and Food Safety Laboratory, Beltsville MD, United States of America; 2 Maryland Institute for Applied Environmental Health, University of Maryland School of Public Health, College Park, MD, United States of America; 3 Department of Plant Science and Landscape Architecture, University of Maryland, College Park, MD, United States of America; 4 Department of Animal and Food Sciences, University of Delaware, Newark, DE, United States of America; 5 Department of Agriculture and Resource Sciences, University of Maryland Eastern Shore, Princess Anne, MD, United States of America; University of Campinas, BRAZIL

## Abstract

Irrigation water contaminated with *Salmonella enterica* and *Listeria monocytogenes* may provide a route of contamination of raw or minimally processed fruits and vegetables. While previous work has surveyed specific and singular types of agricultural irrigation water for bacterial pathogens, few studies have simultaneously surveyed different water sources repeatedly over an extended period of time. This study quantified *S*. *enterica* and *L*. *monocytogenes* levels (MPN/L) at 6 sites, including river waters: tidal freshwater river (MA04, n = 34), non-tidal freshwater river, (MA05, n = 32), one reclaimed water holding pond (MA06, n = 25), two pond water sites (MA10, n = 35; MA11, n = 34), and one produce wash water site (MA12, n = 10) from September 2016—October 2018. Overall, 50% (84/168) and 31% (53/170) of sampling events recovered *S*. *enterica* and *L*. *monocytogenes*, respectively. Results showed that river waters supported significantly (p < 0.05) greater levels of *S*. *enterica* than pond or reclaimed waters. The non-tidal river water sites (MA05) with the lowest water temperature supported significantly greater level of *L*. *monocytogenes* compared to all other sites; *L*. *monocytogenes* levels were also lower in winter and spring compared to summer seasons. Filtering 10 L of water through a modified Moore swab (MMS) was 43.5 (Odds ratio, p < 0.001) and 25.5 (p < 0.001) times more likely to recover *S*. *enterica* than filtering 1 L and 0.1 L, respectively; filtering 10 L was 4.8 (p < 0.05) and 3.9 (p < 0.05) times more likely to recover *L*. *monocytogenes* than 1L and 0.1 L, respectively. Work presented here shows that *S*. *enterica* and *L*. *monocytogenes* levels are higher in river waters compared to pond or reclaimed waters in the Mid-Atlantic region of the U.S., and quantitatively shows that analyzing 10 L water is more likely recover pathogens than smaller samples of environmental waters.

## Introduction

Cases of foodborne illness related to contaminated fruits and vegetables have focused attention on the microbial quality of agricultural inputs, like irrigation water and biological soil amendments. Irrigation water has been implicated as the source of two separate outbreaks of *Escherichia coli* O157:H7 infections associated with Romaine lettuce in the U.S. leading to the deaths of 5 individuals and sickening 272 individuals [1,2]. Bacterial pathogens like *Salmonella enterica* and *Listeria monocytogenes* can contaminate fruits and vegetables because produce is largely consumed minimally processed or raw, without undergoing sufficient antimicrobial treatment to eliminate pathogenic contamination present. *Salmonella enterica* subsp. *enterica* serovar Newport has contaminated a variety of commodities like cucumbers and tomatoes grown in the Mid-Atlantic region of the United States, which have caused outbreaks in the past [3,4]. *L*. *monocytogenes* was responsible for one of the deadliest foodborne outbreaks in U.S. history when 147 individuals fell ill and 33 died from eating contaminated cantaloupe in 2011 [5]. Recent work has shown that *S*. Newport can survive and potentially grow in simulated agricultural runoff [6]which may be present in river waters used for irrigation, and be introduced through runoff to soils amended with biological amendments and transfer to growing spinach plants in pre-harvest environments [7]. The potential presence of *L*. *monocytogenes* in surface irrigation waters can contaminate melons, and if introduced to cantaloupes, can survive at high levels depending on post-harvest storage temperature [8].

Several investigators have shown that *S*. *enterica* and *L*. *monocytogenes* can be present in river and irrigation waters in different regions of the U.S. [9,10,11,12]. If these water sources are used for irrigation of produce crops without disinfection to reduce the level of these pathogens, it is possible for water from freshwater (creeks, rivers, ponds) to contaminate produce intended for human consumption. As stated in the *Standards for the Growing*, *Harvesting*, *Packing*, *and Holding of Produce for Human Consumption*, the U.S. Food and Drug Administration has proposed monitoring irrigation water quality used on farms using levels of *Escherichia coli* (the Produce Safety Rule) [13]. FDA proposes the geometric mean of tested samples where water will contact the edible portion of the crops is not to exceed 126 colony forming units (CFU) of generic *E*. *coli* per 100 mL. The statistical threshold value (STV) for the same samples over time, which must not exceed 410 CFU of generic *E*. *coli* in 100 mL of water.

Quantifying levels of *E*. *coli* can be a useful indicator of fecal pollution in recreational waters to limit viral and gastrointestinal illness of human in direct contact with marine waters [14]; however, other work has shown that *E*. *coli* levels are poor indicators of other bacterial pathogens like *S*. *enterica* and *L*. *monocytogenes* [15,16]which are of concern in agricultural waters. Therefore, other bacterial pathogens may need to be directly quantified or detected to appropriately determine their prevalence. Previous work examining 400 samples taken from 20 agricultural ponds located on farms on the eastern shore of Virginia showed that *Salmonella* were present at low levels (4.4 MPN/100mL) [11]. Other examinations of ponds located on 14 farms in the Mid-Atlantic region showed that 7.7% (3/39) of pond water samples contained *Salmonella* [17]. However, these studies only examined ponds and not other sources of water (freshwater creeks, reclaimed water). A previous study found that the prevalence of *Salmonella* in stream/pond/water/sediment samples was significantly greater than from other categories of pre-harvest samples (fruit, native vegetation, insects, feces, farm soil, and irrigation water) [18]. Previous studies in the Mid-Atlantic region of the U.S. has focused on determining prevalence and levels of *Salmonella* spp and *E*. *coli* because of their public health and regulatory importance, respectively. While recent studies have examined the prevalence of shiga-toxigenic *E*. *coli* (STEC) in various Mid-Atlantic water sources [19], little attention has been paid to levels of *L*. *monocytogenes* in Mid-Atlantic rivers, ponds or reclaimed water. Studies evaluating the presence of *E*. *coli* or *Salmonella* have routinely focused on agricultural ponds or creeks, but few have evaluated all of these water types simultaneously on the same dates over an extended period of time. As variable climate conditions affect the quality and availability of agricultural water, non-groundwater sources of irrigation water (river water, reclaimed wastewater, ponds, produce washwater) warrant attention for their use in agricultural irrigation. However, an assessment of the microbial quality of these water types, along with physicochemical factors which may be associated with differing levels of prevalence, is needed to understand the specific hazards posed by their use as irrigation sources.

The objectives of this study were to quantify levels of *Salmonella enterica* and *L*. *monocytogenes* in various water sources which could be used for irrigation of produce crops over a two-year period. This work was also conducted to determine if volume of water analyzed from these various sources affected the likelihood of recovering either *S*. *enterica* or *L*. *monocytogenes*.

## Materials and methods

### Site description

Six sites in the Mid-Atlantic U.S. were included in this study to represent conventional water sources (rivers, ponds) which are commonly used, and those which could be used in the future (reclaimed wastewater, produce washwater), for agricultural irrigation. During the months in the fruit and vegetable growing season (September–October 2016, June–October 2017, May–September 2018), samples were collected twice a month from each location. During months in the non-growing seasons (November 2016 –May 2017, November 2017 –April 2018, October 2018), samples were taken only once a month. The six water sites and the number of sampling events (n) included: one tidal freshwater river (MA04, n = 34), one non-tidal freshwater creek, (MA05, n = 32), one reclaimed water holding pond (MA06, n = 25), two pond water sites (MA10, n = 35; MA11, n = 34), and one produce wash water (MA12, n = 10). Both river sites (MA04, MA05) are proximate to farms that use surface water for irrigation; ponds (MA10, MA11) were located on university research farms and have been used for irrigation purposes. Reclaimed water (MA06) was used for either groundwater recharge or was disinfected before further processing. Produce washwater was water used throughout vegetable processing within the facility and then applied to soils for irrigation. Table 1 shows description of the water source, the seasons, and number of samples in which the samples were taken. No specific permissions were required because sites were available by public access or on university campuses. No field sampling sites affected any endangered or protected species.

**Table 1:** Description of sites and number of surface or reclaimed water samples taken by season, Fall 2016–2018.

Sites	Description		Catchment area	Spring	Summer	Fall	Winter
MA04	Tidal freshwater river flowing into Choptank River		Marshland/ forested	7(20.6%)	10(29.4%)	11(32.4%)	6(17.6%)
MA05	Non-tidal freshwater river, tributary of Patuxent River		Forested, with grass on shore lime	7(21.9%)	9(28.1%)	10(31.2%)	6(18.8%)
MA06	Reclaimed water treated by grinding, activated sludge processing, secondary clarification, and stored in open air lagoon		--	6(24%)	10(40%)	9(36%)	N/A^1^
MA10	Freshwater pond with depth of ca. 3.35 m and surface area of 0.26 ha		Agricultural	7(20%)	10(28.6%)	12(34.3%)	6(17.1%)
MA11	Freshwater pond with depth of ca. 3 m and surface area of 0.40 ha.		Agricultural	7(20.6%)	10(29.4%)	12(35.3%)	5(14.7%)
MA12	Produce wash water—from enclosed holding tank that may contain reclaimed produce wash water and runoff from cement pad and surrounding area		--	N/A^1^	7(70%)	3(30%)	N/A^1^

^1^ No water samples available for microbial analysis from MA06 in winter or from MA12 in Spring and Winter.

### Water sample collection

Water samples were collected by using a modified Moore swab (MMS) composed of grade #90 cheesecloth (Lions Services, Inc., USA) and rolled into a cylinder 16 cm long with a 4 cm diameter, based on a design previously described by Sbodio et al. *[20]*. Several of the sites (Table 1) were the same sites analyzed for shiga-toxigenic *Escherichia coli* (STEC) reported previously [19]. The MMS was autoclaved and aseptically inserted into a polyvinyl chloride (PVC) cartridge that had been disinfected by soaking in 10% commercial hypochlorite solution (bleach) overnight (modified from Sbodio et al.). Cartridges were rinsed in sterile water after disinfection to remove residual hypochlorite. The cartridge contained a male barbed insert to allow for connection to a water pump via suction hose. On the opposite end was a mesh screen, which served as a debris filter upstream of the swab and to lock the cartridge into a flotation device. The floatation device (also made of PVC) was developed and utilized to collect samples from a depth of 15 cm while also minimizing the risk of agitating sediment in water bodies. Overall, 168 and 170 sampling events were taken during this study, resulting in 504 and 510 separate water samples analyzed for the presence of *Salmonella* spp. and *L*. *monocytogenes*, respectively.

At each site for each sampling event, 10 L, 1 L, and 0.1 L of water were filtered through MMS in triplicate (for a total of nine MMS). For each 10 L sample using the collection device described above, water was actively pumped (Honda, Model # WX10TA, USA) through a single MMS. Samples from the reclaimed water (MA06) were collected from spigots close to field release points. For each 10 L sample, water was first allowed to run for 1 minute prior to water collection. A sterile carboy was then used to collect 20 L, and collected water was treated with 20 mL of a 10% sodium thiosulfate solution (ST; Alfa Aesar, Tewksbury, MA, USA) to neutralize any potential free-chlorine in the water. Following this treatment, water was filtered as described above, and MMS were collected for analysis. For the collection of each 1 L and 0.1 L samples from each surface (river and pond) site, water was collected by immersing an inverted sterile graduated cylinder into the water body, turning it upright to collect water, and then gravity-filtering either 1 L or 0.1 L of water through the MMS. For the reclaimed water site, 1 L and 250 mL were collected from the spigot in sterile bottles, treated with 1 mL and 250 μL of ST, and 1 L and 0.1 L were gravity filtered as described above, respectively (sample collection performed in triplicate). Following filtration, the MMS were transferred to a sterile Whirl-pak bag (Nasco, Fort Atkinson, WI, USA) and placed in a cooler on ice for transportation back to the laboratory. Three MMS for each volume (10 L, 1 L, and 0.1 L) were taken on each sampling day. In most cases, samples from all six sites were collected on the same day.

The following physiochemical parameters were measured using EXO2 or ProDSS multiparameter water quality sonde/meter (YSI, Yellow Springs, OH, USA) for each site: water temperature (^o^C) dissolved oxygen (mg/L), electrical conductivity (μS/cm), pH, oxygen-reduction potential (ORP) (mV), turbidity (FNU), and salinity (PSU). Nitrate was measured using the YSI nitrate sensor. For river and pond water sites, terrestrial and weather conditions including cloud cover, type of onshore vegetation, bank condition (slope/amount of vegetation), and tidal cycle (retrieved from the Maryland Department of Natural Resources) were recorded on a descriptive basis. Furthermore, cumulative precipitation amounts up to 1, 7, and 14 days prior to sampling and ambient temperature were retrieved from Weather Underground (www.wunderground.com). Similarly, for the wastewater reclamation sites, cloud cover, wastewater influent source(s), and the type of primary, secondary, and tertiary treatments were recorded.

### Recovery of *Salmonella enterica* and *Listeria monocytogenes* from Modified Moore Swabs

To each Whirl-pack bag containing a MMS, 100 mL of Universal Pre-enrichment Broth (UPB) (Accumedia, Lansing, MI, USA) was added and hand massaged for 1 min. Enriched MMS in UPB were then incubated at 37°C overnight (18–24 h). After incubation, the sample bags were massaged for 1 minute, and 40 mL of UPB enrichment was aseptically transferred into a sterile 50-mL conical tube. To determine the presence of *Salmonella*, enrichment liquid was vortexed and 1 mL and 0.1 mL were transferred into tubes containing 9 mL of tetrathionate (TT) broth (Accumedia) and 10 mL of Rappaport-Vassiliadis (RV) broth (Accumedia), respectively, which were then incubated overnight at 42°C. For analysis for the presence of *L*. *monocytogenes*, 1 mL of enriched UPB was transferred to a tube containing 10 mL Buffered Listeria Enrichment Broth (BLEB; Accumedia) supplemented with 0.1% sodium pyruvate (Sigma-Aldrich, St. Louis, MO, USA) and incubated at 37°C for 24 h. Selective enrichment broths (1 μL) for *Salmonella* and *L*. *monocytogenes* were isolated on to XLT4 (Accumedia) and RAPID’L.mono (BioRad, Hercules, CA, USA) agar, respectively, and incubated at 37°C for up to 48 h.

Presumptive colonies of *Salmonella* and *L*. *monocytogenes* were re-isolated on respective selective media. DNA from these isolates was obtained using the InstaGene Matrix DNA extraction kit (Bio-Rad), following manufacturer instructions with the following modification: a single colony was transferred directly to a sterile 1.5 mL microfuge tube containing InstaGene Matrix, rather than first being suspended in water and pelleted by centrifugation and supernatant removal. DNA extracts were stored at -20°C, until ready for real-time PCR assay for confirmation of presumptive isolates. A multiplex real time PCR assay specific for *Salmonella* and *L*. *monocytogenes* was used for confirmation of these isolates [21]. Assays were conducted on an Mx 3005P QPCR system (Agilent, Santa Clara, CA, USA) using the SensiFAST Probe Lo-ROX kit (Bioline, Memphis, TN, USA) with an initial denaturation of 10 min at 95 ^o^C, 40 cycles of 20 sec at 95 ^o^C, 30 sec at 64 ^o^C, and 30 sec at 72 ^o^C, and with a final extension of 7 min at 72 ^o^C. Primer and probe (Sigma-Aldrich; Integrated DNA Technologies, Coralville, IA, USA) sequences can be found in Table 2.

**Table 2:** Primers and probes used in multiplex real time PCR assay for a) confirmation of presumptive *Salmonella* spp and *L*. *monocytogenes* based on Kawasaki et al. (2010).

Oligonucleotide	Sequence (5'→3')	PCR Amplicon size	Target Organism
TS-11	GTCACGGAAGAAGAGAAATCCGTACG	375 bp	*Salmonella* spp.
TS-5	GGGAGTCCAGGTTGACGGAAAATTT		
S-FAM	[FAM]ACAAGAAGCCCTGAGCGCCGCTGTGAT[BHQ1]		
LM1	CGGAGGTTCCGCAAAAGATG	234 bp	*Listeria monocytogenes*
LM2	CCTCCAGAGTGATCGATGTT		
L-HEX	[HEX]AGTTCAAATCATCGACGGCAACCTCGGA[TAM]		

As stated previously, three MMS at each volume (10 L, 1 L, 0.1 L) were analyzed, yielding a total of nine MMS for each sampling date. *Salmonella* and *L*. *monocytogenes* populations at each site and date of collection were quantified using a Most Probable Number (MPN) assay at three different volumes 10 L, 1L, and 0.1 L using a freeware MPN calculator software “MPN Calculator” (http://www.i2workout.com/mcuriale/mpn/, Mike Curiale, Build 23, VB6 –link no longer active).

### Statistical analysis

For culture-based recovery of *Salmonella* and *L*. *monocytogenes*, MPN values were calculated. The MPN assay had a minimum limit of detection (LOD) of < 0.03 MPN/L, while the maximum detection limit was 11 MPN/L. For MPN values which were at the LOD, a value of 0.015 (LOD/2) was used for calculation, while 11 MPN/L was used as the value for those samples which had exceeded the maximum detection limit.

**Fig 1:**
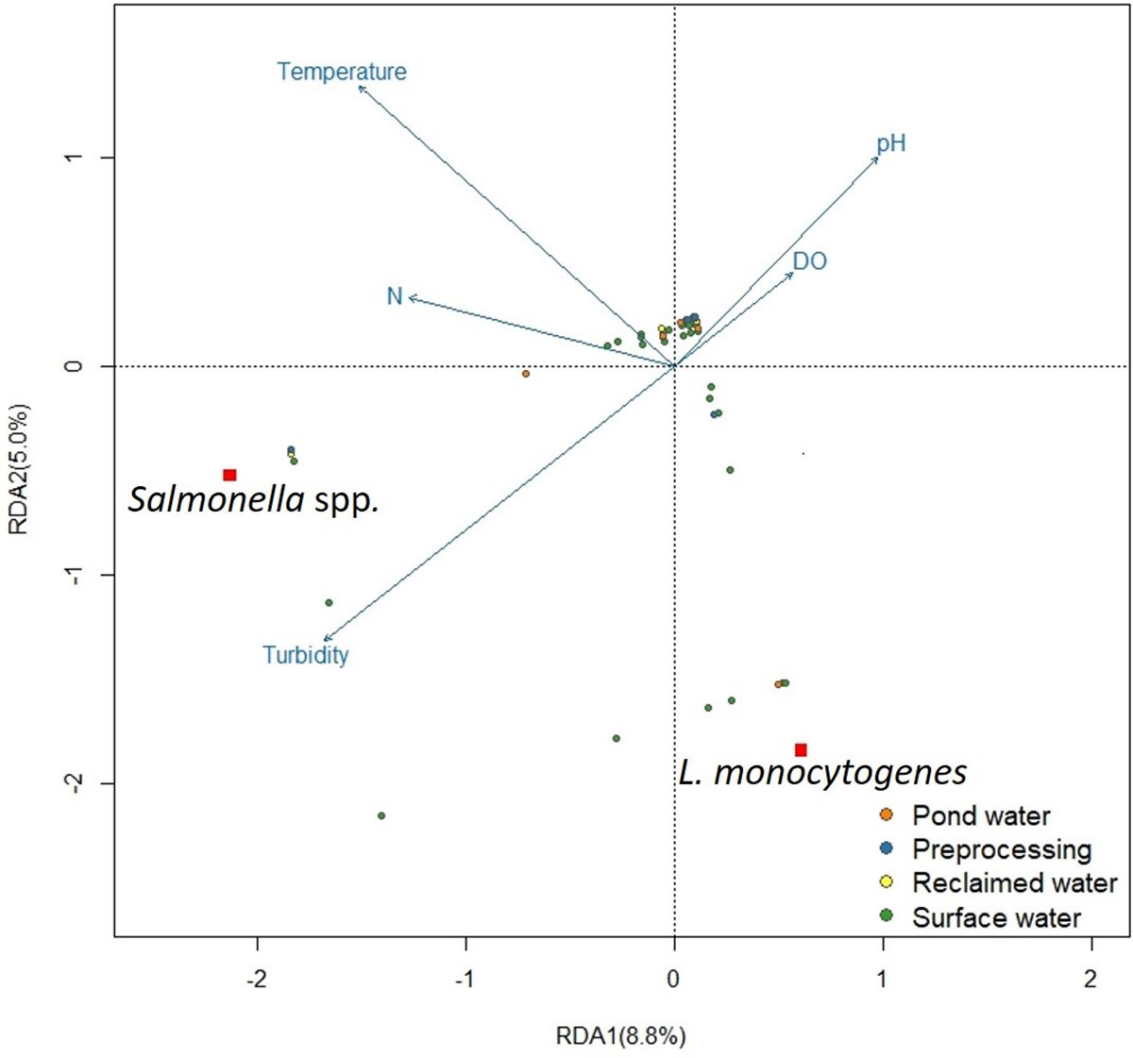
The total constrained variance (13.8%) is plotted on the x-axis (RDA 1 –eigenvalue 8.8%) and y-axis (RDA2 –eigenvalue 5.0%). The direction of the environmental characteristic (blue arrows) points to the direction of increase of that characteristic. Shorter distances between dots (*Salmonella* spp., *L*. *monocytogenes*, water types) and water quality (blue arrows) indicate higher levels of that characteristic relative to other categorical (water types) or response (pathogen) variables.

A redundancy analysis (RDA) in R [22,23] was performed on 137 water samples, two pathogens (*S*. *enterica* and *L*. *monocytogenes*), and eight physicochemical factors: water temperature (^o^C) dissolved oxygen (mg/L), electrical conductivity (μS/cm), pH, oxygen-reduction potential (ORP), turbidity (FNU), salinity (PSU) and nitrate-nitrogen (N). An analysis of variance using permutation evaluated the constraining variables (environmental characteristics) and categorical values (water types) to determine their significance in the RDA model. Water temperature, pH, nitrogen, dissolved oxygen, and turbidity were determined to be significant (p < 0.10) factors in relation to *S*. *enterica* and *L*. *monocytogenes* levels (response variables). For the RDA analysis, the constrained and unconstrained variance were determined to be 13.8% and 86.2%, respectively. The constrained variance was used to make RDA plots to visualize ordinate relationships between *Salmonella* and *L*. *monocytogenes* levels and eigenvectors of significant environmental characteristics. Results from RDA analysis (Fig 1) were used to identify specific factors and areas of statistical analysis described below.

A log transformation was performed for MPN values before analysis of variance (ANOVA). ANOVA was performed by using a mixed effects (lme4) model with multicomp package in R [24,25] with site as the random effect and season and water type as the fixed effects. A pairwise Tukey test (p < 0.05) was used to compare differences in *Salmonella* and *L*. *monocytogenes* log MPN/L values between different seasons (winter, spring, summer, fall), and water types (river, pond, reclaimed, and produce wash), and different sites. In order to compare means of *Salmonella* and *L*. *monocytogenes* MPN/L by site, the MA05 river site was used a reference and a linear regression was performed using the variables site and season.

To compare the recovery of each pathogen by filtration volume, a binomial logistic regression was performed by constructing a general linear mixed effect model in R (glmer)[24]. The fixed effects of the models were season and the volume (categorical variables) while the random variable was the site. The model was applied to each replicate sampling volume on dates when either *Salmonella* or *L*. *monocytogenes* was recovered. The dependent variable was setup as a binary decision {1,0} when {1} indicates the agreement between either the detection or lack of detection of the pathogen in a specific volume (0.1L, 1L, and 10L) compared to the other two volumes assayed on that day; and {0} indicates the disagreement between the same conditions. Filtered volumes across sites were included. Odds ratios determined the probability of recovery of the pathogens by volume collected, and Tukey’s test was used to determine if recovery probabilities comparing the different filtered volumes were significant (P < 0.05).

## Results

The RDA plot (Fig 1) shows ordinal relationships between *Salmonella* and *L*. *monocytogenes* with the constrained variance of the environmental characteristics. The total constrained variance (13.8%) is represented by the eigenvectors RDA1 and RDA2. This is the proportion of the *S*. *enterica* and *L*. *monocytogenes* variance which can be accounted for by water physiochemical factors. Fig 1 shows that when all water types are evaluated overall, levels of *Salmonella* are positively correlated with increasing nitrate-nitrogen and turbidity values but negatively correlated with dissolved oxygen and pH values. *L*. *monocytogenes* values are negatively correlated with increasing water temperature and total nitrogen values. As presented in Fig 1, most of the water samples, regardless of type, are clustered around the center of the RDA ordination graph; however, several river water samples have high positive correlations with both *S*. *enterica* and *L*. *monocytogenes*. The results from the RDA analysis pointed to further investigation of *S*. *enterica* levels in river waters compared to other types of water, and to the association of *L*. *monocytogenes* in river waters with either lower temperatures or seasons with lower temperatures.

**Fig 2:**
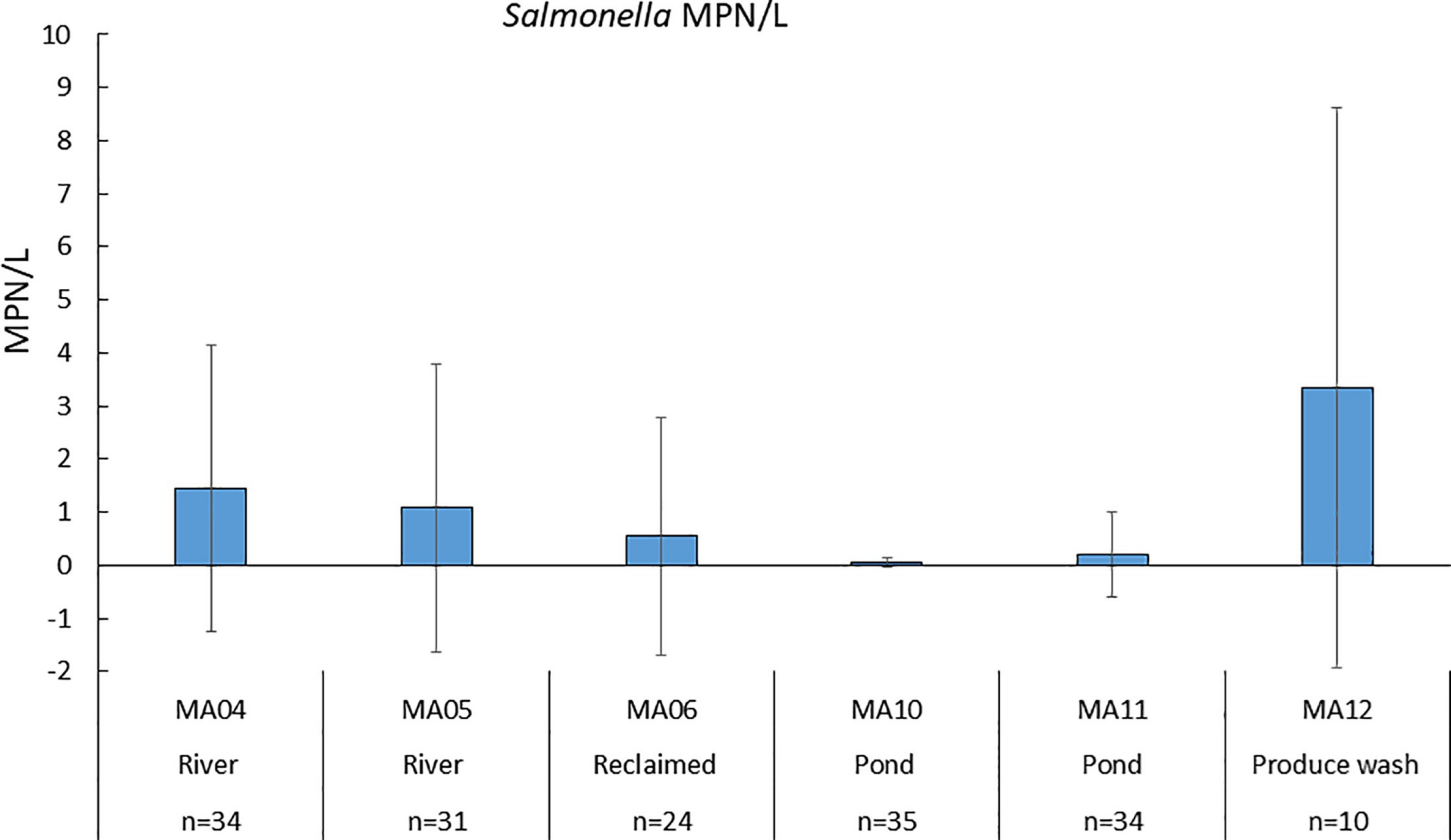
Mean MPN/L values for *Salmonella* for all six sites (n = number of samples) where water samples were taken from Fall 2016–2018.

**Fig 3:**
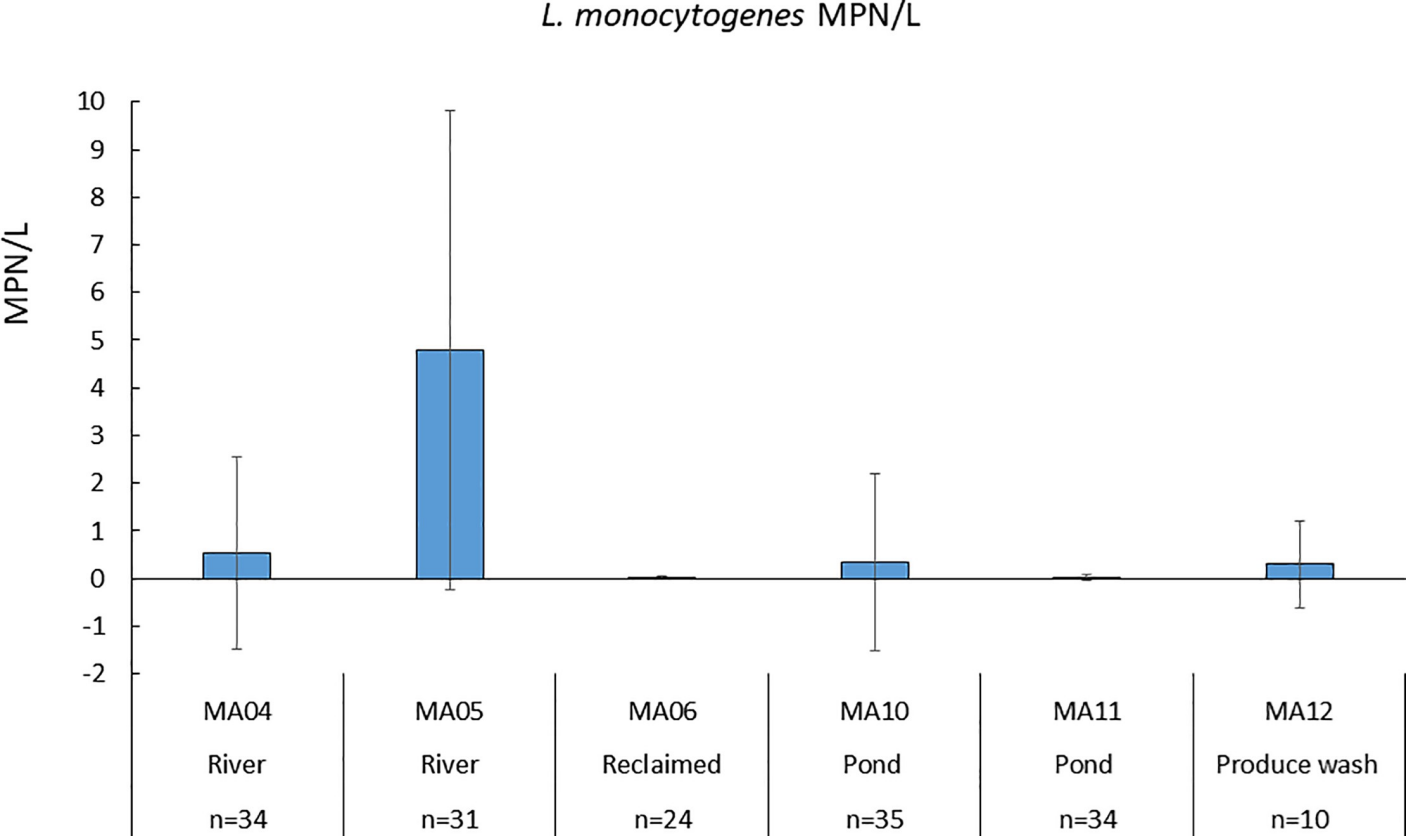
Mean MPN/L values for *L*. *monocytogenes* for all six sites (n = number of samples) where water samples were taken from Fall 2016–2018.

Overall, 50% (84/168) and 31% (53/170) of sampling events recovered *S*. *enterica* and *L*. *monocytogenes*, respectively, from all sampling sites and dates. Fig 2 showed greater MPN/L values for *Salmonella* in river water sites MA04 (1.44 ±2.70 MPN/L) and MA05 (1.08± 2.70 MPN/L) compared to pond water sites MA10 (0.04±0.08 MPN/L) and MA11 (0.20±0.79 MPN/L) levels, supporting findings that river waters had significantly higher levels of *Salmonella* compared to pond water. Reclaimed water also had significantly lower levels of *Salmonella* (0.54±2.24 MPN/L) compared to river water. Levels of *Salmonella* were significantly greater (p < 0.01) in river water compared to pond water, and significantly (p < 0.001) lower in pond water compared to produce wash water. Similarly, *Salmonella* levels in reclaimed waters were significantly (p < 0.001) lower than in river water. There were no significant seasonal differences (p > 0.05) between *Salmonella* levels. Results for *L*. *monocytogenes* are shown in Fig 3. Unlike with *Salmonella*, levels of *L*. *monocytogenes* were significantly (p < 0.05) higher in winter compared to summer, and significantly (p < 0.05) higher in spring compared to summer. Comparing *L*. *monocytogenes* prevalence at different sites, MA05 (river water) site had significantly (p < 0.001) greater *L*. *monocytogenes* levels compared to the five other sites, including the other river water site (MA04). Water temperatures were significantly lower at MA05 compared to other sites by 3–6°C when controlling for the effect of seasons.

Recovery percentages of *Salmonella* and *L*. *monocytogenes* in 0.1 L, 1 L, and 10 L volumes filtered through MMS are shown in Table 3. To calculate the Odds ratio of recovery of pathogen by filtration volume, only sampling events (days) where a positive result for either *Salmonella* or *L*. *monocytogenes* were used. Filtered volumes of 10 L led to a significantly (p < 0.001) greater likelihood—a 43.5-fold increase—for the recovery of *Salmonella* when compared to filtering 0.1L. Similarly, filtering 10 L compared to 1 L led a significantly (p < 0.001) greater likelihood—a 25.5-fold increase–of recovering *Salmonella*. There was no significant (p > 0.05) increase in the likelihood of recovering *Salmonella* when filtered volumes of 1 L vs 0.1 L were compared. For the recovery of *L*. *monocytogenes*, filtering 10 L of water compared to 0.1 L significantly (p = 0.012) improved the chances of detecting the pathogen by 4.8-fold. Filtering 10 L vs 1L volumes significantly (p = 0.037) improved the likelihood of recovery of *L*. *monocytogenes* by 3.9-fold. As with *Salmonella*, there was no significant difference in the likelihood of recovery of *L*. *monocytogenes* when filtering 1 L vs 0.1 L. These results indicate that when optimizing recovery methods of enteric foodborne pathogens in potential irrigation waters, filtering 10 L compared to 1L or 0.1L enhances the recovery of bacterial pathogens.

**Table 3:** Number (percentage) of total sampling events at each site where each water volume filtered contained *Salmonella* or *L*. *monocytogenes*.

			*Salmonella* spp.				*L*. *monocytogenes*		
Site	Water type	Number of sampling events	0.1 L	1 L	10 L		0.1 L	1 L	10 L
MA04	River	34	17 (50%)	16 (47.1%)	27 (79.4%)		9 (26.5%)	6 (17.6%)	14 (41.2%)
MA05	River	32	8 (25%)	15 (46.9%)	25 (78.1%)		25 (78.1%)	29 (90.6%)	29 (90.6%)
MA06	Reclaimed	25	2 (8%)	5 (20%)	8 (32%)		2 (8%)	2 (8%)	2 (8%)
MA10	Pond	35	1 (2.9%)	2 (5.7%)	7 (20%)		2 (5.7%)	2 (5.7%)	3 (8.6%)
MA11	Pond	34	2 (5.9%)	4 (11.8%)	10 (29.4%)		1 (2.9%)	2 (5.9%)	3 (8.8%)
MA12	Produce wash	10	5 (50%)	4 (40%)	6 (60%)		1(10%)	1 (10%)	1 (10%)

## Discussion

The occurrence of outbreaks of salmonellosis and listeriosis associated with fruit and vegetable commodities consumed raw or after minimal processing has focused attention on the presence of bacterial pathogens in irrigation water used in agricultural production. Data presented here shows that *S*. *enterica* and *L*. *monocytogenes* levels differed by water types, with lower levels of the pathogens present in pond water sites and higher levels present in river waters. Our study attempted to collect and analyze water on the same days to achieve some temporal consistency among the sample analysis. The redundancy analysis showed that the majority of the variance (86.2%) was not constrained, indicating that it could not be attributed closely with the eight physicochemical characteristics measured in all waters. This analysis is in agreement with previous studies which have shown poor correlation between the ability of physicochemical factors to influence levels of *S*. *enterica* in river waters in Florida [26]. Other authors have shown that *Salmonella* levels in ponds are not correlated to total suspended solids (TSS) before or after rainfall events [27]. Prevalence rates of *Salmonella* (50%) and *L*. *monocytogenes* (31%) in the current study are greater than those reported for STEC (2.35%) using several of the same sampling dates and sites [19].

Table 3 shows the percentage of *Salmonella*-positive samples recovered from 10 L filtered volumes from river water sites ranged from 78.1–79.4%, which was greater than those from pond water—20–29.4%—and from reclaimed water (20%). Surface waters may be contaminated with inputs containing *Salmonella*, which could include runoff from manure applied to agricultural field and storm runoff, where bacterial pathogens may accumulate in the waterway [27]. *Salmonella* levels were reported to be 1-log higher after storm events than before the event in streams and ditches in Georgia [27].

*Salmonella* prevalence in surface waters vary around the US. In California river waterways, 65% (908/1405) of samples taken from lakes, streams, rivers, and ponds contained *S*. *enterica*, and overall prevalence was significantly lower in fall than in spring or summer [10]. Cooley et al. [10] left Moore swabs in water bodies overnight before they were collected and analyzed for *Salmonella*. In a separate California study, 7.1% (18/252) of water samples contained *S*. *enterica* [28]when grab water samples were obtained. *S*. *enterica* were detected in 79% (57/72) of river waters taken from the Suwanee river basin in a coastal plain in Georgia [15]. In North Carolina, 55% (47/84) of water samples taken contained *S*. *enterica* [29]. These regional variations in *Salmonella* prevalence may be related to animal husbandry activities near watersheds, rainfall and storm events, untreated wastewater, and temporal and spatial variability in these waters.

Pond water (MA10, MA11) sites in our study were both located on research farms and used for irrigation. In the Mid-Atlantic, the prevalence of *Salmonella* in agricultural ponds has received much attention because of the potential persistence of *S*. Newport linked to several outbreaks originating from this region [3]. Our work reports lower levels of *Salmonella* in ponds compared to river waters. Previous investigators found that 19% (38/200) of sampling events at 20 ponds on farms over two years on the eastern shore of Virginia contained *Salmonella* when 250 mL of a 1 L sample were analyzed [11]. In our study, 5.7% (2/35) and 11.8% (4/34) of sampling events at MA10 and MA11 pond sites, respectively, contained *Salmonella* when 1 L was filtered through MMS. However, when analyzing 10 L volumes which were filtered, *Salmonella* prevalence increased to 20% and 29% for MA10 and MA11, respectively. Water samples collected for filtration in ponds were collected from banks of the pond. Other research has shown that location of water collection from within pond sites can affect the quantitative recovery of *Salmonella* and *E*. *coli* [30,31].

*L*. *monocytogenes* was prevalent in 31% (53/170) of sampling events in this study. *L*. *monocytogenes* was reported in 10% (32/314), 45% (15/33), and 43% (604/1405) of samples taken in Ontario, Canada, New York state and California, respectively [10,12,32]. Other studies have shown a prevalence rate of 28% of *L*. *monocytogenes* in river water samples in New York state and 18% in rural and urban watershed samples in Nova Scotia, Canada [16,33]. In results reported in the current study, temperature affected the level and prevalence of *L*. *monocytogenes*. A greater percentage of 10 L samples were positive for *L*.*monocytogenes* at the MA05, the site with the lowest mean water temperature, compared to all other sites. *L*. *monocytogenes* levels were significantly greater in spring compared to summer, and in winter compared to summer as well. Previous studies have also shown that *L*. *monocytogenes* prevalence was higher in winter (72%) compared to spring (59.1%) or summer (55.4%) [16]. These results also somewhat agree with those found by Cooley et al. [10], who showed that *L*. *monocytogenes* prevalence was lower in fall months than in winter or spring months. *L*. *monocytogenes* is considered a psychrotroph which may explain higher prevalence rates and levels in colder months.

Lower levels of both *S*. *enterica* and *L*. *monocytogenes* (Figs 2 an d3; Table 3) recovered from reclaimed water compared to river water sites are not surprising since it has undergone physical and chemical treatments to remove contaminants (Table 1) [34].Reclaimed water was analyzed from a holding pond after treatment, which may have allowed low levels of *Salmonella* and *L*. *monocytogenes* to be introduced to the pond through animal intrusions or soil runoff. Other studies did not find the presence of *Salmonella* or *L*. *monocytogenes* in reclaimed wastewater but used non-culture-based methods and smaller volumes in their detection methods [35]. Lower prevalence of *Salmonella* and *L*. *monocytogenes* indicate that the microbial quality of reclaimed wastewater from this site may be suitable for agricultural irrigation. Higher levels of *Salmonella* observed in produce washwater (MA12) are not surprising since water was collected after produce washing and contact with cement surfaces, allowing contaminants to potentially accumulate in water before storage in a holding tank. Other investigators have found a low prevalence of *Salmonella* (0.4%) and *L*. *monocytogenes* (0.7%) in spinach washwater [36].

Levels and prevalence of *L*. *monocytogenes* in our study may have been affected by the recovery methodology used. In our study, we used a 24-h enrichment of MMS in Universal Pre-enrichment Broth (UPB), as opposed to a more commonly used 4 h non-selective enrichment before addition of selective supplements or transfer to a selective broth. In our case, UPB enrichments were placed into Buffered Listeria enrichment broth (BLEB) after 24 h. Previous work attempting to recover pathogens from environmental samples on dairy farms showed that the use UPB had similar recovery rates of *L*. *monocytogenes* as the selective *Listeria* Enrichment Broth (LEB) [37]. Our recovery of *L*. *monocytogenes* may have been influenced by the presence of other *Listeria* spp., along with other types of bacteria, which may have competed with the pathogen in the non-selective UPB [16].

Table 4 shows that *Salmonella* and *L*. *monocytogenes* recovery from all water sources was significantly enhanced when filtering 10 L versus 1 L or 0.1 L through a MMS for culture analysis. Many of the other studies reported on above used different methods to collect water, and most only collected a single volume of water; quantification in those studies was achieved by diluting water from that single sample [11]. Our study quantified the likelihood of recovering bacterial foodborne pathogens from collecting or filtering different volumes of the source water. Filtering 10 L of agricultural water through a MMS may not be as sensitive as recovering pathogens as ultra-filtration methods [38], but may facilitate taking multiple samples from different locations on the same day of sample collection A previous study in Maryland, filtering 10 L of river water through a MMS similar to the method described here, isolated *Salmonella enterica* from 65% (30/46) of river water samples taken from four separate sites (*9*). A larger percentage of samples from rivers in Sinaloa, Mexico were shown to contain *Salmonella* (76%) when 10 L was ultra-filtered (UF) than when 1 ml aliquots were assayed (44%) [39]. Other studies using ultra-filtration of 20 L of surface water showed that 45% (48/107) of samples contained *Salmonella* spp. [40].While it is likely that the MMS cheesecloth filter has a lower recovery efficiency than ultra-filtration through hollow fiber filters for bacterial pathogens [38], MMS can be used in a MPN assay since each filter, similar to each tube in a 9-tube MPN in the laboratory, only requires evaluation for the presence or absence of the target pathogen. The utilization of 10 L combined with MMS provided a cost-effective tool with a minimum of field equipment to survey for bacterial foodborne pathogens from numerous water sites. These data are useful to show that even at sites or water bodies which have low levels of *S*. *enterica* and *L*. *monocytogenes*, collecting and filtering 10 L of water may improve the likelihood of detection.

**Table 4:** Odds ratios of recovery of *Salmonella* and *L*. *monocytogenes* by comparison of different filtration volume. P-values less than 0.05 are significant.

Pathogen	Filtered Volumes compared	Odds Ratio	p-value
*Salmonella* spp.	1L VS 0.1L	1.7	0.194
	10L VS 0.1L	43.5	<0.001
	10L VS 1L	25.5	<0.001
*L*. *monocytogenes*	1L VS 0.1L	1.2	0.894
	10L VS 0.1L	4.8	0.012
	10L VS 1L	3.9	0.037

Results presented here show that the overall prevalence of *Salmonella* is greater than that of *L*. *monocytogenes* in several ponds and rivers in the Mid-Atlantic region. *Salmonella* levels were more prevalent in river water compared to ponds or reclaimed water sites, while *L*. *monocytogenes* levels were greater in colder seasons and at river water sites which had colder temperatures. Overall, this study represents one of the few studies evaluating *L*. *monocytogenes* presence in the Mid-Atlantic region of the US, and provides quantitative data to show that collecting and filtering 10 L is more likely to detect target pathogens than smaller volumes, even when they are present at consistently low levels. These results can be used to provide information on when to use non-groundwater sources for irrigation of fruits and vegetables, and inform irrigation practices that reduce the transfer of foodborne pathogens from water to produce crops.

## Supporting information

S1 File *Salmonella* and *L. monocytogenes* MPN and water quality parameters. Salmonella and L. monocytogenes populations (MPN/L) with water quality parameters for all data analyzed for Fig 1, Fig 2 and Fig 3.
(XLSX)

S2 File CONSERVE Culture OR. Dataset used to calculate the OR (odds ratio) between detection of either Salmonella or L. monocytogenes by filtration volume.
(XLSX)
